# Broad reverse approach surgery for hidradenitis suppurativa (BRASH): An inside-out surgical technique for localized hidradenitis suppurativa

**DOI:** 10.1016/j.jdin.2026.05.021

**Published:** 2026-06-06

**Authors:** Giulio Gualdi, Davide Bertolla, Fabrizio Panarese, Ludovica Ucci, Patrick Silvetti, Leonardo Balestra, Giuditta Trobbiani, Paolo Amerio

**Affiliations:** a University Hospital, Ospedale Maggiore di Parma, Parma, Italy; b Section of Dermatology and Infectious Diseases, Department of Medical Sciences, University of Ferrara, Ferrara, Italy; c Dermatologic Clinic, G. D'Annunzio University, Chieti, Italy

**Keywords:** hidradenitis suppurativa, inside-out approach, localized disease, reconstruction, surgical technique, tissue-sparing surgery

## Challenge

Hidradenitis suppurativa is a chronic, recurrent inflammatory disease of the follicular unit characterized by painful nodules, abscesses, tunnels, and scarring in intertriginous areas. Although medical therapy is essential to control inflammation, surgery is often required for persistent localized tunnelized lesions unlikely to resolve pharmacologically. Standard surgical approaches, most commonly deroofing or excision, typically address the disease from the surface inward.^1^^,^^2^ In selected cases requiring wider excision and reconstruction, skin grafts or dermal substitutes may be needed, increasing complexity, costs, and resource use. These considerations support simple, reproducible, tissue-sparing approaches for carefully selected localized lesions.

## Solution

We describe broad reverse approach surgery for hidradenitis suppurativa (BRASH), a simple inside-out surgical technique for selected localized hidradenitis suppurativa lesions. After delineation of the involved area, the affected skin and subcutaneous tissue are elevated as an adequately thick dermo-subcutaneous flap, including sufficient underlying fat. The diseased block is then reflected like opening a book, exposing the deep surface of the flap and allowing the lesion to be approached from beneath rather than from the skin surface. With the tissue temporarily turned over, curettage and sharp excision are performed under direct visualization, selectively removing macroscopically diseased tissue, fibrotic bands, fistulous tracts, and tunnelized extensions. Excessive flap thinning is avoided, and broad lateral attachments are preserved whenever possible to maintain vascularity. The aim is to clear the adnexal-bearing diseased substrate while preserving viable tissue for direct closure. A step-by-step demonstration of the procedure is provided in Video 1, available via Mendeley at https://data.mendeley.com/datasets/pjr39kssbc/1. In our practice, BRASH is performed with lidocaine without adrenaline/epinephrine, so that bleeding points are not masked by vasoconstriction and can be controlled before closure. The surgical bed and undersurface of the flap are carefully inspected, irrigated, and closed only after meticulous hemostasis. Acute abscesses, extensive purulence, or uncontrolled infection should be treated before considering this approach. BRASH is intended for carefully selected localized lesions with limited tunnel formation, manageable fibrosis, mobile surrounding tissue, and feasibility of low-tension primary closure. It is not intended for extensive confluent disease, broad interconnected tunnels, severe deep fibrosis, or lesions requiring grafting or complex reconstruction. In our clinical experience across selected patients and anatomical sites, no local recurrence or major complications were observed during extended follow-up. To our knowledge, this specific inside-out application to hidradenitis suppurativa has not been previously reported.

## Conflicts of interest

None disclosed.
